# Interkingdom interaction between *C. albicans* and *S. salivarius* on titanium surfaces

**DOI:** 10.1186/s12903-020-01334-w

**Published:** 2020-12-01

**Authors:** Loyse Martorano-Fernandes, Nadiny Cezar Rodrigues, Maria Heloísa de Souza Borges, Yuri Wanderley Cavalcanti, Leopoldina de Fátima Dantas de Almeida

**Affiliations:** 1 Postgraduate Program in Dentistry, Federal University of Paraíba, Cidade Universitária, João Pessoa, Paraiba Brazil; 2 School of Dentistry, Federal University of Paraíba, Cidade Universitária, João Pessoa, Paraiba Brazil; 3 Department of Clinic and Social Dentistry, Federal University of Paraíba, Cidade Universitária, João Pessoa, Paraiba Brazil

**Keywords:** Peri-implantitis, *Candida albicans*, *Streptococcus salivarius*

## Abstract

**Background:**

In oral candidiasis models, *Candida albicans* and *Streptococcus salivarius* sp. biofilms have an antagonistic relationship. Due to this, *S. salivarius* have been used experimentally as probiotic. However, the interaction between these microorganisms in the peri-implantitis-like microenvironment remains unknown. This study aimed to evaluate the interaction between *C. albicans* and *S. salivarius* biofilms developed on titanium surfaces, under reduced oxygen levels.

**Methods:**

Titanium specimens were pre-conditioned with artificial saliva (1 h, 37 °C). Single-species biofilms of *C. albicans* (ATCC 90028) and co-culture biofilms of *C. albicans* and *S. salivarius* (ATCC 7073) was developed for 24 and 72 h on titanium specimens. Subsequently, the effect of these intervals of biofilm formation and the interactions among the cells were evaluated. Biofilms from cultures were collected and analyzed for cell viability (CFU/mL), biofilm biomass, and total protein dosage. Data were analyzed using Mann–Whitney test (α = 5%). In addition, co-culture biofilms were analyzed using fluorescence microscopy.

**Results:**

*C. albicans* growth did not change due to the presence of *S. salivarius*. Besides, co-culture biofilms showed a significant difference in the number of viable cells between 24 and 72 h of biofilm development (*p* < 0.05). The highest biofilm biomass and protein dosage were observed in co-cultures at 72 h of biofilm development. Fluorescence microscopy showed that co-cultures biofilms at 24 h have limited number of pseudo-hyphal and hyphae cells of *C. albicans*. At 72 h, these types of cells have increased. *S. salivarius* in both stages of development was present in some clusters surrounded by *C. albicans*.

**Conclusions:**

Co-cultivation of *C. albicans* with *S. salivarius* in biofilms developed on titanium surfaces, under lower oxygen levels, did not affect fungus growth. In addition, *S. salivarius* did not hind *C. albicans* virulence. These findings suggest that the use of *S. salivarius* as a probiotic would be ineffective in peri-implant disease treatment.

## Background

Prostheses supported by titanium dental implants are commonly used for the rehabilitation of total and partially edentulous patients [1]. However, approximately 22–43% of cases develop inflammatory diseases called peri-implant mucositis and peri-implantitis, which occur mainly due to the presence of biofilm [2]. These diseases begin when microorganisms interact with each other, attach on the titanium implant surface and proliferate, reaching a mature biofilm capable to invade the tissue and induce an inflammatory process in the host [3]. In advanced stages of those diseases, the bone around the implant reabsorb, which can result in the loss of the dental implant [4–6]. Thereby, understanding the biofilm formation and the microorganism’s interactions could create targeted approaches to pathogen control.

The peri-implant biofilm is mostly composed of *Candida albicans* [7, 8], which has the ability to form robust mixed biofilms and invade tissues [9, 10]. In the peri-implant region, *C. albicans* establishes interactions with *Streptococcus* species, which can benefit (synergism relationship) or inhibit (antagonism relationship) the fungal growth, modulating the potential of *C. albicans* to cause diseases [10]. In an oral candidiasis model, a known antagonism relationship occurs between *C. albicans* and *Streptococcus salivarius*, through a metabolic product known as bacteriocin-like inhibitory substances (BLIS). This metabolic is released extracellularly by the bacteria and can kill or interfere with the growth of pathogenic microorganisms [11, 12]. Due to this mechanism of action, *S. salivarius* may be used experimentally as probiotic to treat oral candidiasis [11, 13].

However, the relationship between *C. albicans* and *S. salivarius* under peri-implantitis-like microenvironment is still unclear. Therefore, the purpose of this in vitro study was to evaluate the interaction between *C. albicans* and *S. salivarius* biofilms developed on titanium surfaces, under reduced oxygen levels. To understand this interaction, single-species biofilms of *C. albicans*, single-species biofilms of *S. salivarius* and co-cultures of *C. albicans* and *S. salivarius* were developed on the surface of pre-conditioned titanium specimens. After 24 h and 72 h, the biofilms were analyzed regarding cell viability, biomass quantification, and total protein dosage. Altogether, our results suggest that *C. albicans* – *S. salivarius* interactions under peri-implant environment might be different from other oral conditions; and these findings showed new directions regarding the treatment of the peri-implant disease.

## Methods

### Specimens’ preparation

Standardized discs of commercially pure titanium (1.3 × 0.2 cm) were prepared according to with previously methodologies [14]. The specimens did not have any treatment on their surface. Therefore, there was not any antimicrobial activity or potential to inhibit the biofilm formation. The titanium discs were cleaned with 70% alcohol (v/v) and sterilized by autoclave at 121 °C for 15 min prior to use.

### Microbial strains and growth conditions

*C. albicans* (ATCC 90028) and *S. salivarius* (ATCC 7073) were used to generate single-species and co-culture biofilms. These strains were reactivated aerobically from their original cultures in Agar Sabouraud Dextrose (Difco, Detroit, USA) and Brain Heart Infusion (Kasvi, Italy) at 37 °C, respectively. The yields of microorganisms were analyzed by seeding bacterial and fungi suspension into agar plates. Therefore, the concentration of microorganisms was based on CFU/mL quantification. Three to five colonies of each strain were collected and suspended in 5 mL of sterile saline (0.9% NaCl). Then, cells were centrifuged (5000 g for 5 min), washed twice with saline, and suspended in RPMI 1640 medium (Inlab diagnóstica, Brazil) to standardize concentrations. The concentration of *C. albicans* at OD_600_ was 1.0 × 10^6^ CFU / mL, whilst the concentration of *S. salivarius* was 1.0 × 10^8^ CFU/mL (LGL Scientific 0741/16, Brazil). These concentrations were based on experiments described previously [14, 15]. RPMI 1640 was used because has the nutritional requirements of both microorganisms [16, 17].

### Preconditioning with artificial saliva and biofilm development

Initially, titanium discs were immersed in 500 µL of artificial saliva composed of 1% carboxymethyl (w/v); 0.0084% sodium chloride (w/v); 0.12% potassium chloride (w/v); 0.0342% potassium phosphate (w/v); 0.0146% calcium chloride (w/v), and 0.052% magnesium chloride (w/v) [18, 19]; following by incubation at 37 °C for 60 min [20]. Subsequently, the specimens were randomly and individually allocated into 24-well plates. For single-species biofilms, each titanium disc was inoculated with *C. albicans* or *S. salivarius*. For co-culture biofilms, both *C. albicans* and *S. salivarius* were added to the inoculum. The plates were incubated at 37 °C under a micro-aerobic atmosphere using an anaerobic jar with a candle, which reduced the presence of oxygen, similar to a peri-implantitis-like microenvironment [20]. The culture medium was changed daily until the end of the experimental period (72 h). The development of each biofilm was assessed at 24 h (mature biofilm) and 72 h (biofilm dispersion stage). Biofilms were analyzed with regards the number of colony-forming units (CFU/mL), biomass quantification, and total protein dosage. The experiments were performed independently in duplicate (n = 12/group).

### Cell viability analysis

After 24 h and 72 h, specimens were transferred to microtubes containing 1.0 mL of sterile saline and agitated in a vortex for 60 s [20]. Subsequently, the suspensions were serially diluted to determine the number of viable microorganisms (10^−1^ to 10^−6^). Aliquots of 10 µL from each dilution were seeded in Sabouraud Dextrose Agar (SDA) and Mitis Salivarius Agar (MSA). The plates incubated at 37 °C for 24 h. The number of viable cells was determined after counting colony-forming units. The values were multiplied by the serial dilution and converted to a logarithmic scale, expressed in CFU/mL.

### Biomass quantification

The biofilms’ biomass quantification was performed using the crystal violet assay [21]. Culture medium was removed from the plates and the discs were dried for 45 min at 37° C. Afterwards, 600 µL of crystal violet aqueous solution (Labsynth Produtos para Laboratório LTDA, Diadema, Brazil) at 0.5% (w/v) was added on discs for 30 min. The solution was removed and washed three times with sterile saline. Subsequently, 600 µL of 70% acetic acid (v/v) was added. The supernatant was read using spectrophotometry at 590 nm.

### Total protein dosage

The measurement of total proteins was performed according to the biuret assay [22], using a commercial diagnostic kit for total proteins (Labtest, Minas Gerais, Brazil). The calibration solution used had 4 g/dL bovine albumin and 14.6 mmol/L sodium azide. Initially, the culture medium was removed from the plates and the discs washed with 500 µL of sterile saline. The solution was centrifuged (5000 g for 5 min) and added 500 µL of 1 M NaOH. After that, the samples were vortexed and centrifuged for 10 s. For cell lysis, 1.0 mL of biuret reagent was added. The samples were incubated for 10 min at 37 °C. Afterwards, the absorbance of samples was read in a spectrophotometer at 495 nm. Based on the obtained data, the total protein was calculated considering the sample absorbance by the calibration factor.

### Fluorescence microscopy

Co-culture biofilms developed during 24 h and 72 h were analyzed descriptively using fluorescence microscopy (Leica Microsystems GmbH, Wetzlar, Germany). Representative images were generated to assess interactions between bacteria and fungi, as well as the *C. albicans* cell types (yeast-form cells, oval pseudo-hyphal cells, and elongated hyphal cells) [14]. Previously the analysis, biofilms were fixed with 10% (v/v) formal-saline for 48 h at 4 °C. The samples were then stained with 10 μL of propidium iodide (25 μM; Molecular Probes, Paisley, UK) to stain *S. salivarius* and 10 μL of calcofluor white (1% (v/v); Sigma-Aldrich) to stain *C. albicans* [14]. Representative images (100 μm × 100 μm) of both dye-channels were obtained from five different fields of view. *C. albicans* morphotypes analysis was based on previous investigations parameters [14].

### Statistical analysis

The Statistical Package for Social Sciences (SPSS) software (SPSS, IBM, Chicago, IL, USA) was used for data analysis. Data were analyzed with regards their normality (ShapiroWilk test) and homoscedasticity (Levene test). Statistical analysis was performed using Mann–Whitney test with 5% significance (α < 0.05) and minimal power of 80%. In all experiments, two statistical analyses were performed. Firstly, the development stage of biofilm was considered the comparison factor. Mature (24 h) and dispersion stage (72 h) biofilms were compared to understand the effect of these time intervals on biofilm formation. Then, second analysis compared the biofilm type (sigle-species or co-culture) to comprehend the meaning of interactions among the cells within the same interval (24 h or 72 h).

## Results

**Fig. 1:**
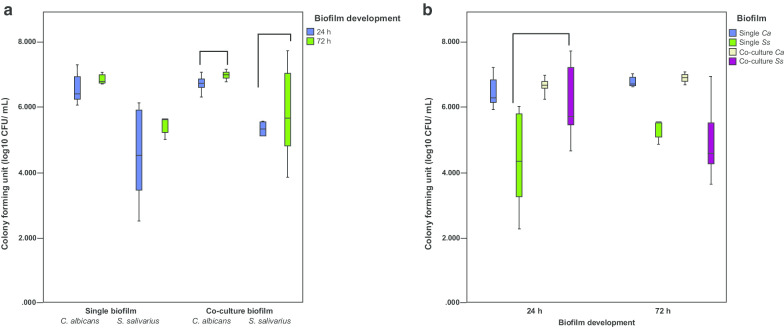
Cell viability of single-species and co-culture biofilms (n = 12). **a** Development stage of biofilms at 24 h and 72 h. Co-culture biofilms of *C. albicans* and *S. salivarius* showed a statistically significant difference between 24 and 72 h of biofilm development compared to single-species biofilms (*p* < 0.05). Connected groups present a statistical difference (Mann–Whitney, *p* < 0.05). Data shown are from box-plot: each box contains 50% of the group data; the lower and upper limits represent the 25th and 75th percentiles, respectively; bars represent the minimum and maximum values, and the horizontal line inside the box represents median. **b** Biofilm types (single-species or co-culture biofilms) within the same time interval (24 h or 72 h). Cell viability of *S. salivarius* at 24 h differed statistically between single-species and co-culture biofilms. (*p* < 0.05). Connected groups present a statistical difference (Mann–Whitney, *p* < 0.05). Data shown are from the box-plot: each box contains 50% of the group data; the lower and upper limits represent the 25th and 75th percentiles, respectively; bars represent the minimum and maximum values, and the horizontal line inside the box represents median

Single-species and co-culture biofilms of *C. albicans* presented higher number of viable cells within 24 h and 72 h compared to those of *S. salivarius*. Overall, single-species biofilms of both species did not change significantly between 24 and 72 h. In contrast, co-culture biofilms showed a significant difference between 24 and 72 h of biofilm development (*p* < 0.05) (Fig. 1a).

To better understand the effect of cell physical contact between *C. albicans* and *S. salivarius* we evaluated the cell viability of each biofilm type. Interestingly, *C. albicans* did not have its viability changed in single-species and co-culture biofilms. On the other hand, the higher number of viable cells of *S. salivarius* was detected in co-culture biofilms at 24 h. Moreover, the growth of *S. salivarius* at 24 h was significantly different between single-species and co-culture biofilms (*p* < 0.05) (Fig. 1b).

**Fig. 2:**
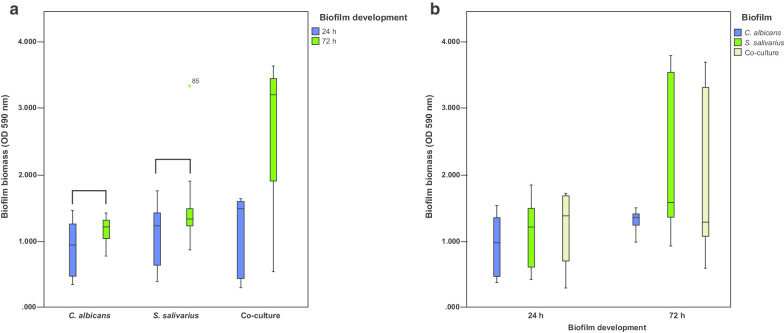
Biofilm biomass of single-species and co-culture biofilms (n = 12). **a** Development stage of biofilms at 24 h and 72 h. Single-species biofilms of *C. albicans* and *S. salivarius* showed statistically significant difference compared to co-culture biofilms at 24 h and 72 h (*p* < 0.05). Connected groups present a statistical difference (Mann–Whitney, *p* < 0.05). Data shown are from box-plot: each box contains 50% of the group data; the lower and upper limits represent the 25th and 75th percentiles, respectively; bars represent the minimum and maximum values, and the horizontal line inside the box represents median. **b** Biofilm types (single-species or co-culture) within the same time interval (24 h or 72 h). The groups did not differ statistically (*p* > 0.05). Connected groups present a statistical difference (Mann–Whitney, *p* < 0.05). Data shown are from the box-plot: each box contains 50% of the group data; the lower and upper limits represent the 25th and 75th percentiles, respectively; bars represent the minimum and maximum values, and the horizontal line inside the box represents median

Regarding the biofilm biomass, co-culture biofilms presented higher amounts of cells at 72 h. Although this happened, it was not enough to be significant, as long as single-species biofilms of *C. albicans* and *S. salivarius* showed a significant difference between 24 and 72 h of biofilm development, compared to co-culture biofilms (*p* < 0.05) (Fig. 2a). Overall, single-species and co-culture biofilms of *C. albicans* and *S. salivarius* did not show a significant difference in biofilm biomass (*p* > 0.05) (Fig. 2b).

**Fig. 3:**
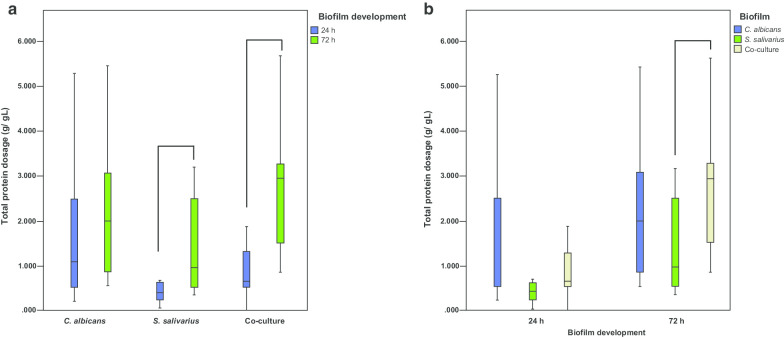
Total protein dosage (n = 12). **a** Development stage of biofilms at 24 h and 72 h. Single-species biofilms of *S. salivarius* showed a significant difference at 24 h and 72 h compared to single-species biofilms of *C. albicans* (*p* < 0.05)*.* Co-culture biofilms also demonstrated a significant difference between 24 and 72 h (*p* < 0.05). Connected groups present statistical differences (Mann–Whitney, *p* < 0.05). Data shown are from box plot: each box contains 50% of the group data; the lower and upper limits represent the 25th and 75th percentiles, respectively; bars represent the minimum and maximum values, and the horizontal line inside the box represents median. **b** Biofilm types (single-species or co-culture) within the same time interval (24 h or 72 h). Single-species biofilms of *S. salivarius* and co-culture biofilms at 72 h presented statistically significant differences (*p* < 0.05). Connected groups present a statistical difference (Mann–Whitney, *p* < 0.05). Data shown are from the box-plot: each box contains 50% of the group data; the lower and upper limits represent the 25th and 75th percentiles, respectively; bars represent the minimum and maximum values, and the horizontal line inside the box represents median

With regards the total protein production, higher amount of proteins occurred at 72 h of biofilm development for single-species and co-culture biofilms. Single-species biofilms of *S. salivarius* presented significantly higher concentration of total protein (*p* < 0.05). Co-culture biofilms also demonstrated a statistically significant difference between 24 and 72 h (*p* < 0.05) (Fig. 3a). Single-species biofilms of *S. salivarius* presents statistically lower quantity of total protein compared to that observed in co-culture biofilms (*p* < 0.05) (Fig. 3b).

**Fig. 4:**
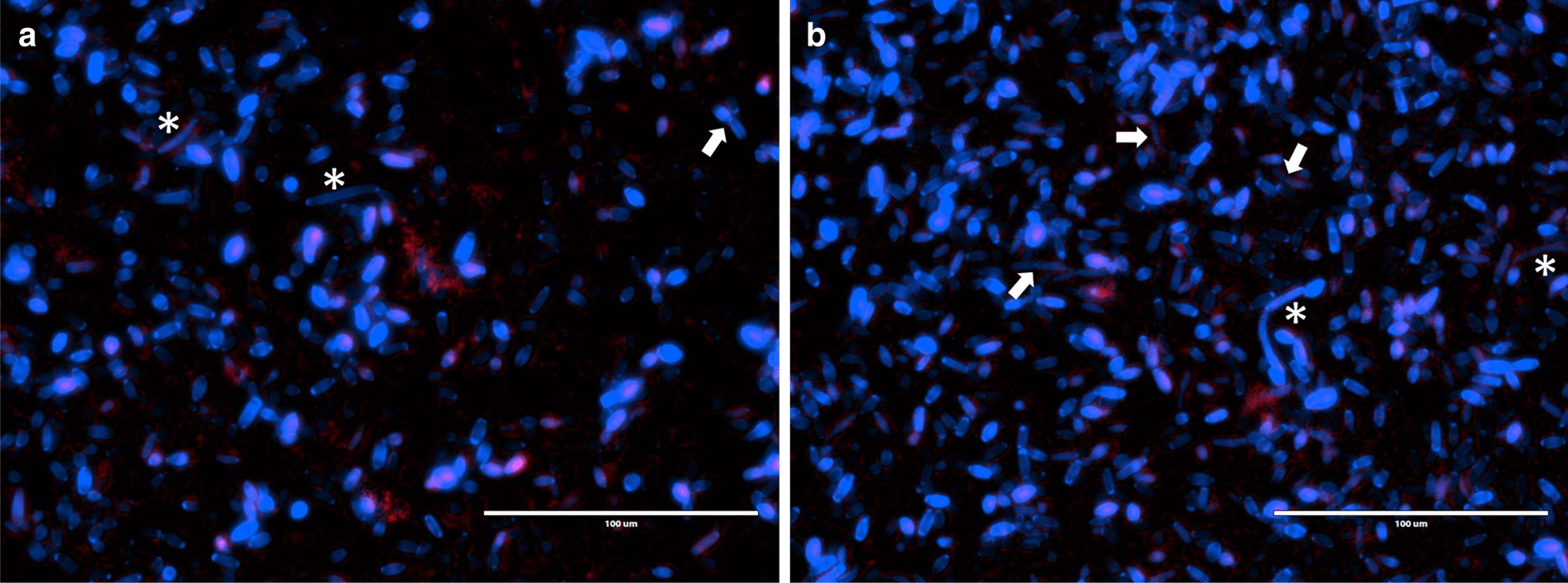
Representative images of fluorescence microscopy of co-culture biofilms of *C. albicans* and *S. salivarius* developed under peri-implantitis-like microenvironment conditions. *C. albicans* is stained with calcofluor white (blue) and *S. salivarius* is stained with propidium iodide (red). Arrows demonstrate *C. albicans* pseudo-hyphal, whilst * shows hyphae cells. **a** Co-culture biofilms at 24 h. Note limited pseudo-hyphal cells and hyphae surrounded by clusters of bacteria. **b** Fungi-bacteria biofilm developed at 72 h. Note numerous pseudo-hyphal cells and some hyphae. Also, the presence of bacteria agglomerates

Fluorescence microscopy has shown that co-cultures biofilms at 24 h presented higher amounts of yeast-form cells surrounded by limited pseudo-hyphal cells and hyphae (Fig. 4a). On the other hand, co-cultures biofilms at 72 h showed numerous pseudo-hyphal cells and some hyphae (Fig. 4b). *S. salivarius* cells at 24 h and 72 h of biofilm formation are presented in some clusters around the fungi (Fig. 4a, b).

## Discussion

Mucositis and peri-implantitis are infections caused by fungi and bacteria biofilms [23–25]. Understanding this biofilm is essential to guide future therapeutic approaches, such as the use of probiotics. Traditionally, in oral candidiasis models, *C. albicans* and *S. salivarius* establish antagonistic relationships, in which the fungal cells are surrounded by *S. salivarius*, resulting in a decrease adhesive of ability and pathogenic potential [11, 13]. Thus, *S. salivarius* may be used as an alternative to the treatment of oral candidiasis. However, our findings suggest the antagonistic relationship *C. albicans* and *S. salivarius* was not established within the peri-implant microenvironment, in which biofilms were developed on titanium surfaces under low oxygen levels. Therefore, the use of *S. salivarius* as a probiotic would not be effective in treating peri-implant diseases.

Previous studies demonstrated an antagonistic interaction between *C. albicans* and *S. salivarius* using the following bacteria strains SK56, DSM14685, and K12 [11, 13, 26–28]. In our study, *S. salivarius* ATCC 7073 was used in all experiments. Evidence shows that the *S. salivarius* NCTC 8618 strain, which is a homologous strain of ATCC 7073 (available from https://www.atcc.org/Products/All/7073.aspx#generalinformation) also develops an antagonism relationship with *C. albicans* tested in vitro and in vivo [29]. These findings reinforce that an antagonistic relationship would be expected between *S. salivarius* and *C. albicans*. Of importance, *S. salivarius* is recognized as a microorganism that does not cause harmful effects on humans and plays an important role in the biofilm composition, by inactivating and establishing antagonistic relationships with oral pathogens, such as *Candida albicans* [11, 13]. Thus, according to our findings, we suggest the antagonistic relationships did not happen due to the conditions in which biofilms were developed.

In the oral cavity, the oxygen levels, the substratum (e.g. mucosa, teeth, prosthesis, and implants), and variations in nutrient content could hind microorganisms to establish interactions [30]. Our study used RPMI 1640 medium, which is known to mimic the composition of human fluids, due to the presence of amino acids such as L-Glutamine, L-Arginine, and L-Asparagine, as well as vitamins and inorganic salts [31]. Previous investigations showed that RPMI 1640 medium can be used to initiate and develop in vitro biofilms of *C. albicans*, similarly to yeast nitrogen base and sabouraud dextrose broth medium [16]. Regarding the bacteria growth, RPMI 1640 has the nutritional requirements of *S. salivarius* [17]. Therefore, RPMI 1640 medium does not hind interactions between the microorganisms tested in this study.

Regarding the cell viability, co-culture biofilms of *C. albicans* showed differences between 24 and 72 h of biofilm development, in which the higher viability was at 72 h. Moreover, *C. albicans* did not have its viability changed in single-species and co-culture biofilms. This data suggested that, under the conditions tested in this study, *S. salivarius* was unable to decrease the number of fungal cells. Observations of *C. albicans* growth indicate that the mature and dispersion stage is mostly composed of cells in a hyphae-form, which are related to virulence and pathogenicity of *Candida* biofilms [32]. At these stages, the dispute for nutrients among the cells may be so high that the bacteria can not interfere with the fungus growth. Although this occurs, the viability of the bacteria has not been disabled. Co-cultures biofilms of *S. salivarius* presented significantly higher number of viable cells.

Interactions between fungi and bacteria occur through physical contact or metabolic products [33–35]. *S. salivarius* can interact with other microorganisms through its bacteriocin-like inhibitory substances (BLIS), which is responsible for maintaining orderly population dynamics within oral microbiota [36]. Although there is a lack of evidence concerning bacteriocin production of the strain *S. salivarius* ATCC 7073, the bacteriocin production has been reported in several *S. salivarius* strains [36–38]. Thus, it might be possible that *S. salivarius* ATCC 7073 also produced bacteriocins because it is a behavior characteristic of the streptococcal species. Notwithstanding, future studies should evaluate the *S. salivarius* ATCC 7073 bacteriocin production.

Despite BLIS contribution to interactions among the microorganisms in an oral candidiasis model, *C. albicans* was not directly inhibited by bacteriocin action. Indeed, physical cell contact is required to inhibit fungi’s growth [11]. These findings suggest that the ability of *S. salivarius* inactivating some microorganisms is apart from the bacteriocin's action. Another explanation for *S. salivarius* did not decreasing *C. albicans* development is the time in which BLIS operates. Usually, microorganisms’ metabolite products act during the early stages of biofilm development, especially during exponential growth [13, 39]. It is possible that at the times evaluated in this study, the metabolite product was inactive, being insufficient to decrease the *C. albicans* growth. Thereby, future studies should evaluate the biofilm at the early stages of development.

In addition, the exponential growth of the *C. albicans* biofilm continues to advance in its maturation stage, resulting in several dense layers of polymorphic cells round in an extracellular matrix. This matrix gives the biofilm a robust and dense structure, which could protect it from chemical and physical injury [40, 41]. One possibility to estimate the contribution of the biofilm’s matrix is by measuring the biomass of the biofilm. Expectedly, single-species biofilms of *C. albicans* have significantly higher biomass at 72 h compared to 24 h. Besides that, our results indicate that co-culture biofilms at 72 h presented higher biomass. These findings suggest that the matrix of biofilm contributes to its architecture and could act as a protective barrier for *C. albicans*.

The extracellular matrix of biofilm has around 500 proteins in its structure, most of which are hydrolyzing enzymes that can disrupt biopolymers as both a protective response and a nutrient source [40]. Preliminarily, we also investigate the proteins in the biofilm cultures throughout the biuret assay. Higher protein production was observed at 72 h of biofilm development, and this is possibly due to the longer period these biofilms remained in cultivation. Moreover, there is possible that a fungi-bacteria relationship and a robust extracellular matrix contributed to increasing the total protein production. However, our results are limited to the dosage of total proteins, which could not estimate which protein would make the greatest contribution to this biofilm. The process of identifying this protein could be important for creating therapeutic targets.

In general, our findings suggest that *C. albicans* growth did not change due to the presence of *S. salivarius*. To better understand the interactions between both microorganisms and the virulence of *C. albicans* through the filamentous formation, fluorescence microscopy was performed. In the early stage (24 h), the yeast cells were prevalent, with few pseudo-hyphal cells and hyphae surrounded by *S. salivarius*. However, at 72 h numerous pseudo-hyphal cells and some hyphae were presented. The filamentous forms of *C. albicans* (hyphae and pseudo-hyphae) are considered pathogenic [42]. Thus, these results suggest that under the conditions tested in this study and during the dispersion stage, *C. albicans* inactivation is a challenge to *S. salivarius*.

Although microscopy evaluations are widely used, future investigations should consider using gene expression analysis or other genetic approaches to evaluate transcriptional regulators of *C. albicans* (Efg1, Tec1, Bcr1, Ndt80, Brg1, and Rob1) [40], virulence factors such as Hwp1 and Als3 [40], as well as invasiveness (Sap family) [43]. In addition, other study designs should consider including relevant bacteria involved with peri-implant infections (i.e. *Porphyromonas gingivalis*) [8] and host–pathogen interactions of *C. albicans* [23].

## Conclusions

Overall, we observed that *C. albicans* development follows its course in phases of development independently of *S. salivarius.* Although *S. salivarius* is a facultative anaerobe, surprisingly, on titanium surfaces and under lower oxygen, the bacterium was not able to inactivate fungal growth. Moreover, at the dispersion stage, *C. albicans* increase their virulence. Therefore, this interaction in the peri-implant environment could be a challenge to *S. salivarius* act as an antagonist microorganism.

## Data Availability

The datasets used and/or analyzed during the current study are available from the corresponding author on reasonable request.

## Abbreviations

ATCC: American type culture collection
CFU/mL: Colony-forming units per milliliter
SDA: Sabouraud dextrose agar
MSA: Mitis salivarius agar
SPSS: Statistical package for social sciences
